# Unit of Analysis Guide, Part 1: More Than a Sum of Parts

**DOI:** 10.1093/asj/sjae123

**Published:** 2024-06-03

**Authors:** Isabella F Churchill, Lucas Gallo, Xi M Zhu, Steven Hanna, Christopher J Coroneos

In 2015, the *Aesthetic Surgery Journal* published a series of Evidence-Based Medicine Special Topics, known as the “EBM Hub,” providing plastic surgeons with a practical overview of research methodology.^1^ The authors also released “EBM Hub Challenges,” educating and challenging readers to critically appraise the methodology and results of a breast surgery article.^2^ In the last decade, the reconstructive and breast surgical fields have contributed the greatest proportion of publications in plastic surgery,^3,4^ with most of these studies assessing surgical outcomes. Despite this, many authors often incorrectly use the breast, rather than the patient, as the unit of analysis. From a statistical standpoint, this has been deemed a definite error.^5^ In this two-part guide we discuss the patient and/or breast as the unit of analysis, critically analyze articles for their unit of analysis, and suggest a tool to provide guidance to surgeons planning studies with 2 symmetric surgical sites (eg, breasts, ears). In Part 1, we describe scenarios in which patients are used as the unit of analysis, outline statistical assumptions and limitations, and critically analyze a clinical trial using the patient as the unit of analysis.

Scenario 1: Imagine a study where researchers randomly assign a total of N patients to receive 1 of 2 surgical treatments (eg, vertical vs Wise pattern). Patients are assessed following surgery and outcomes are collected, which may be a normally distributed score or binary (yes/no). In this case, the researchers want to compare the presence or absence of complications (eg, hematoma) following surgery. The researchers compare the incidence of complications in the treatment vs control groups, considering all the breasts in the dataset, resulting in a sample of 2N breasts. It is found that there is an incidence of 7 hematomas, with 4 hematomas in 2 patients (bilateral hematomas in 2 patients). The correct analysis must not assume independence of the observations by treating them as if they came from 2N different women. To compare rates of complications, we must correctly estimate the variability in complications expected among different women who undergo the same procedure. To the extent that the likelihood of complications is related to patient factors (or the surgeon), then the risk is common to both breasts. Statistically, the test of the difference between women who receive different procedures will be too likely to reject the possibility that there is no difference, if the test assumes independence of the observations. In our example, a Pearson chi-square test of independence will be biased, whereas McNemar's test of paired proportions would be suitable. At the same time, when planning the study, a sample size calculation that proposes to recruit N patients as if they contribute *2*N independent data points will be less powerful than it appears.^6^ As a result, the researchers are likely to recruit too few patients to detect the difference if it exists.

Scenario 2: Now imagine if the researchers redesigned this study and still randomly assign a total of N patients to receive 1 of 2 surgical treatments (eg, vertical vs Wise pattern) and compare the presence or absence of a hematoma following surgery. This time, the proportions of patients with complications in each group are counted. The 7 hematomas are counted and the complications per patient are analyzed and compared between each group. This is a comparison between different patients because each patient contributes 1 outcome to the total sample. Here, the Pearson chi-square test might be suitable because it may be correct to assume independence of each breast,^6^ and various patient-related risk factors are being taken into account (eg, smoking, BMI, and age).^7^ However, it is important to note that there may be other threats to independence in such a study. For example, if the same surgeon treated some patients but not others, there may be common outcomes associated with surgical skill.

Example: In Wall et al, the authors investigated the use of force-modulating tissue bridges for wound closure following elective breast surgery in a case-control study with 122 patients in the intervention group and 121 in the control group.^8^ Inquiries to the authors confirm that there was no matching of patients in the 2 groups on patient factors. The primary outcome was wound healing complications, as measured by breast wounds >3 mm occurring in the vertical limb closure and measured as a dichotomous outcome in each patient (unilateral and bilateral). The authors used the patient as their unit of analysis. It was concluded that there was an 89% reduction in relative risk for a significant wound in the intervention compared with the control group.

Question: Based on the study design and results, which of the following statements, if any, are true?

- “Between-patient” designs require larger sample sizes (using the same confidence and power) to determine an effect estimate compared with a “within-patient” design.
- Using the patient as the unit of analysis did not violate the assumption of independence.
- Results in this study were interpreted correctly as the outcome was measured at the level of the patient, with the outcome being reported per patient.
- Between-patient variance was appropriately accounted for in this study.

Answer: (a), (b), (c), and (d) are all correct.

In Part 1, we demonstrate when it is appropriate to use the patient as the unit of analysis. Given that data in breast surgery research must consider both treatment and patient factors, researchers must first determine what unit of observation (measurement) and unit of analysis (outcome based on sample) their study aims to investigate prior to designing and beginning their analysis. However, in any case, it is important to note that different analyses can provide different conclusions, with some of them providing errors. A common assumption in statistics is the independence of observations. However, dependent data are very common in practice. If Wall et al had matched each intervention patient to a control patient, based on their similarity, this may have induced a dependence within the matched pairs, similar to the effect of using pairs of breasts.^8^ This is commonly seen in clinical studies as there is more than one aspect of pairing/clustering with regards to dependence. Ignoring dependency between observations may result in biased tests.^6,9^ As such, we caution researchers when analyzing and drawing conclusions based on their study results to avoid statistical pitfalls. In Part 2, we will discuss using the breast as the unit of analysis.
